# A Clinical Analysis of Pharyngeal Bronchogenic Cysts in the Pharynx of Children

**DOI:** 10.3389/fped.2021.629009

**Published:** 2021-05-20

**Authors:** Ying Xu, Fugen Han, Dongjie Seng, Lan Jiang, Shengcai Wang, Xin Ni, Jie Zhang

**Affiliations:** ^1^Department of Otorhinolaryngology Head and Neck Surgery, Henan Children's Hospital, Zhengzhou Children's Hospital, Children's Hospital Affiliated of Zhengzhou University, Zhengzhou, China; ^2^Department of Otolaryngology Head and Neck Surgery, Beijing Children's Hospital, National Center for Children's Health, Capital Medical University, Beijing, China

**Keywords:** bronchogenic cysts, pharynx, pathology, treatment, children

## Abstract

**Objective:** This study was designed to summarize the clinical characteristics, diagnosis and treatment of pharyngeal bronchogenic cysts in children to help in making the correct diagnosis and developing an appropriate treatment plan.

**Methods:** The clinical data of 13 children with bronchogenic cysts in the pharynx, who were treated in otolaryngology head and neck surgery department between September 2013 and July 2019, were analyzed retrospectively. The clinical characteristics were evaluated, and the related factors for diagnosis and treatment were analyzed. Clinical characteristics and imaging features of three cases whose lesions located in the nasopharyngeal, oropharynx, and laryngopharyngeal were demonstrated.

**Results:** All 13 children were male, the youngest being 4 days old, the oldest 6 years and 6 months, and the median age being 1 year and 4 months. Eight patients were diagnosed during a physical examination, and five patients visited the doctor with different degrees of upper airway obstruction. The mass was located in the nasopharynx in one patient, in the oropharynx in eight patients, and in the laryngopharynx in the other four patients. Computed tomography (CT) scanning, which is helpful for a topical diagnosis, showed a dense homogeneous mass. Electronic nasopharyngoscopy showed cystic masses of different sizes in the pharynx. All the children underwent cyst resection under general anesthesia, and the postoperative pathology result was a bronchogenic cyst. One child was lost to follow-up, but the remaining 12 children were followed up for between 6 months and 6 years, during which no recurrence of a cyst was found.

**Conclusion:** Bronchogenic cysts are a rare cyst of the head and neck, and the most common site of the cyst is the oropharynx. The impact on airway obstruction depends on the location and size of the cyst. CT scanning is of great significance for diagnosis. Surgical treatment should be carried out as soon as possible after diagnosis, as surgery is the most effective way to treat bronchogenic cysts. Follow-ups should be carried out regularly to prevent cyst recurrence.

## Introduction

Bronchogenic cysts are a rare congenital disease resulting from the abnormal development of primitive foregut, and they occur in one or more tissues of the respiratory system. Although the pathogenesis is unknown, the hypothesis of embryo abscission and translocation proposed by Govaerts et al. is generally accepted (1). It suggests that in the early stage of embryo development, the thorax and abdominal cavity are integrated, and the primary tracheobronchial tree is formed in the 5th week. During this period, all types of abnormal germs fall off and migrate, and since the secretions cannot be discharged, a bronchogenic cyst forms at the migration site. Due to the low incidence rate, the existing literature on head and neck bronchogenic cysts consists mostly of single case reports (2–4), and since a bronchogenic cyst in the pharynx is rare, the rates of misdiagnosis and missed diagnosis before surgery are high. Diagnosis mainly depends on intraoperative findings and postoperative pathology. In the present study, the data of 13 cases of bronchogenic cysts in the pharynx were analyzed, and the clinical characteristics and diagnosis and treatment were summarized to improve the preoperative diagnosis rate of the disease and help in improving treatment plans.

## Methods

### Information and Methods

The clinical data of 13 children with a bronchogenic cyst in the pharynx, who were treated in the Department of Otorhinolaryngology Head and Neck Surgery at Henan Children's Hospital, between September 2013 and July 2019, were analyzed retrospectively. The postoperative pathology result for all the patients was a bronchogenic cyst. All the children were male, the youngest being 4 days old, and the oldest 6 years and 6 months, and the median age was 1 year and 4 months.

### Preoperative Assessment and Diagnosis

The lesions can be divided, according to their location, into nasopharynx bronchogenic cysts, oropharynx bronchogenic cysts, and laryngopharynx bronchogenic cysts. Obstruction of the upper airway to varying degrees manifests as nasal obstruction, snoring, respiratory distress, and dyspnea. On enhanced computed tomography (CT), a typical bronchogenic cyst manifests as a single-chambered, different-sized, round or quasi-round cyst. If secondary infection, bleeding and rupture occur, it may cause the compression of surrounding tissues. Cystic masses of different sizes with clear boundaries can also be seen in the pharynx with the use of electronic rhinolaryngoscopy.

### Therapeutic Methods

Before the operations in this study, the location of the bronchogenic cyst was determined based on imaging and electronic nasopharyngoscope. All 13 children underwent a plasma-assisted operation under general anesthesia. Eight of them, who had oropharyngeal cysts, underwent partial resection of the mass, one patient with a nasopharynx (eustachian tube circular pillow) cyst underwent endoscopic partial resection of the cyst via the oral approach, and the remaining four patients, with laryngopharyngeal (glossal root and epiglottic vallecula) cysts, underwent endoscopic-assisted partial resection of the cyst. All the children underwent surgical removal of the part of the cyst that protruded into the pharyngeal cavity (the outer part of the cyst wall remained), that is to say, a partial resection.

The study was conducted in accordance with the Declaration of Helsinki (as revised in 2013) and was approved by the Ethics Committee of the Children's Hospital Affiliated to Zhengzhou University. Since the participants were under the age of 16, written informed consent was obtained from their parents.

## Results

### Characteristics of the Disease and Pathology

All 13 children were male. The primary site of the mass was in the nasopharynx (eustachian cushion) in one child, in the oropharynx in eight children (in the parapharyngeal wall in six children and in the palatine uvula in two children), and in the laryngopharynx in four children (at the root of the tongue in three children and in the epiglottis valley in one child). The postoperative pathology result of all the children was a bronchogenic cyst (see Table 1).

**Table 1:** Clinical characteristics of 13 cases.

**Case number**	**Gender**	**Age**	**Course of disease**	**Site of lesion**
Case 1	Male	6Y 6M	15D	Nasopharynx (right tubal torus)
Case 2	Male	2Y 2M	2M	Pharyngeal (left lateral pharyngeal wall)
Case 3	Male	4Y 11M	20D	Pharyngeal (left lateral pharyngeal wall)
Case 4	Male	6M 15D	3M	Pharyngeal (right lateral pharyngeal wall)
Case 5	Male	4D	4D	Laryngeal (right vallecula epiglottica)
Case 6	Male	1Y	3M	Pharyngeal (uvula root)
Case 7	Male	10M 30D	4M	Pharyngeal (right lateral pharyngeal wall)
Case 8	Female	1M 6D	20D	Base of tongue
Case 9	Male	2Y 3M	1Y	Pharyngeal (left lateral pharyngeal wall)
Case 10	Male	26D	26D	Base of tongue
Case 11	Male	27D	27D	Base of tongue (vallecula epiglottica)
Case 12	Male	10M 17D	3M	Pharynx oralis (right arcus pharyngopalatinus)
Case 13	Male	8M 22D	3M	Pharyngeal (right uvula root)

*Y, year; M, month; D, day*.

### Clinical Manifestations

Eight patients were diagnosed during a physical examination, while five patients visited the doctor with varying degrees of upper airway obstruction and then underwent electronic nasopharyngoscopy, CT, and Magnetic Resonance Imaging (MRI). Among these children, one child had dyspnea shortly after birth and was treated with mechanical ventilation through endotracheal intubation. When the symptom was alleviated, the ventilator was removed. When dyspnea occurred again, a laryngopharyngeal mass was found during the second intubation. Three children visited the doctor with progressive dyspnea and snoring, and masses were found at the root of the tongue and epiglottis valley. One child snored at night with buccal respiration; examination showed that there was a cystic mass in the eustachian cushion of the nasopharynx.

### Treatment Outcomes

For all the patients, the part of the mass protruding into the cavity of the pharynx was surgically resected (retaining the lateral part of the capsule wall). One child (whose mass was located at the palatine uvula of the oropharynx) was lost to follow-up after the operation, but the remaining 12 children were followed up for between 6 months and 6 years. No recurrence of the cyst was found, there was no snoring or dyspnea, and normal growth and development were observed in all 12 cases during the follow-up period.

## Typical Cases

### Case 1

**Figure 1:**
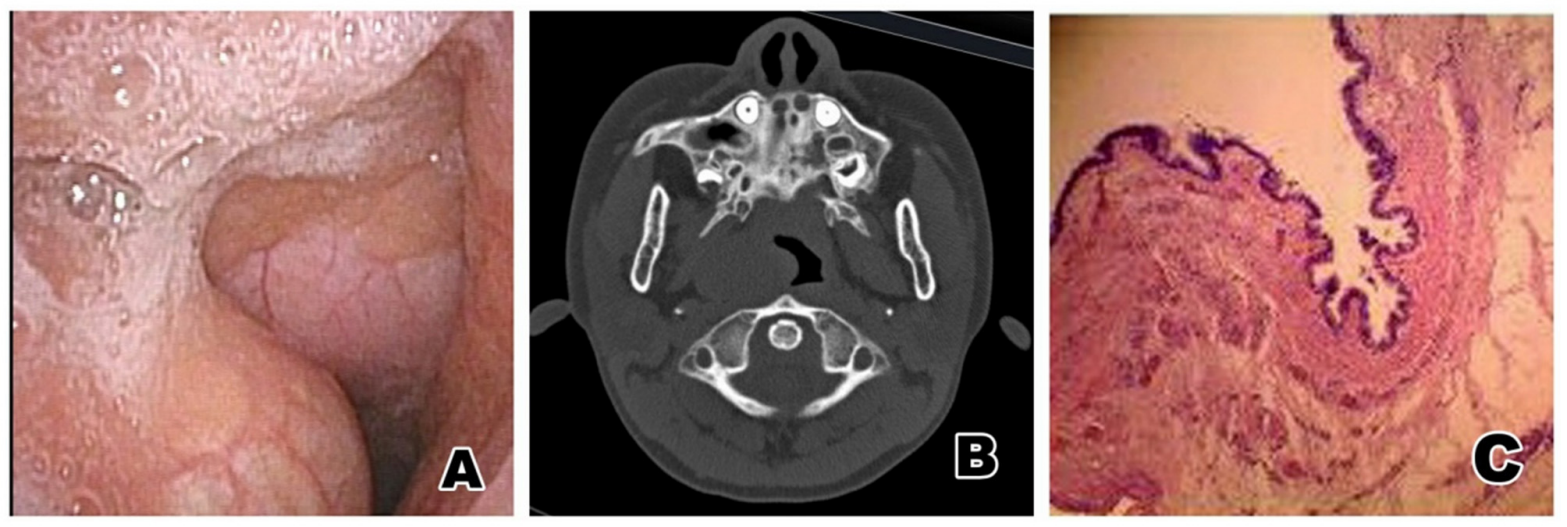
**(A)** Electronic nasopharyngoscopy before operation: A cystic mass of about 2.0 × 2.5 × 1.0 cm with a smooth surface can be seen on the right eustachian cushion in the nasopharynx, extending from the posterior margin of the soft palate to the oropharynx and midline. **(B)** Nasopharynx CT: This image shows a mass-like soft tissue density shadow from the right nasopharyngeal wall to the oropharyngeal wall, the adjacent airway being compressed and narrowed. **(C)** Pathological results: The cyst cavity was lined with squamous epithelium and pseudostratified ciliated columnar epithelium, and smooth muscle and lymphocyte infiltration were seen under the epithelium. The diagnosis was a bronchogenic cyst of the nasopharynx.

The child was a boy aged 6 years and 6 months. He was admitted to hospital due to snoring at night with buccal respiration for 15 days. A physical examination revealed that there was a 1.0 × 1.0 cm mass, protruding from the midline to the left in the right posterior palatopharyngeal arch. The surface of the mass was smooth and soft, and there was no tenderness; no fistula or mass was found in the neck. Electronic nasopharyngoscopy showed that there was a cystic mass of ~2 × 2.5 × 1.0 cm with a smooth surface in the left eustachian cushion, which passed though the posterior margin of the soft palate to protrude into the oropharynx and midline (Figure 1A). Pharynx CT revealed mass-like soft tissue density shadow from the right nasopharyngeal wall to the oropharyngeal wall, and the adjacent airway was compressed and narrowed (Figure 1B). An electro-otoscope was used to show that the bilateral external auditory canals were clean, the tympanic membranes were intact, and the marker was clear. Acoustic immittance exhibited a type A curve, and pure tone audiometry indicated that the hearing in both ears was normal. After a satisfactory level of general anesthesia was achieved, an opener was used, and a catheter was inserted through the double inferior meatus of the nose and through the nasopharynx. It was then taken out through the oral cavity, and the soft palate was pulled up to enlarge the cavity of the pharynx. Endoscopic examination showed that the base of the mass was located on the right eustachian cushion, the size of the mass was ~2 × 2.5 × 1.0 cm, and its distance from the pharyngeal opening of the eustachian tube was ~0.3 cm. The mass was well-exposed, and then punctured, and ~3 ml of light-yellow viscous liquid was extracted. The mass was pulled to the left and down with a clamp through the mouth, and the part of the capsule wall protruding into the cavity of the pharynx was resected with laryngeal scissors and a plasma knife. It could then be seen that the lateral lacuna was depressed outwards, and the surface of the deep capsule wall was smooth, which was not treated. The pathological diagnosis was a bronchogenic cyst (Figure 1C). The child was followed-up for 1 year after the operation, and there was no recurrence.

### Case 2

**Figure 2:**
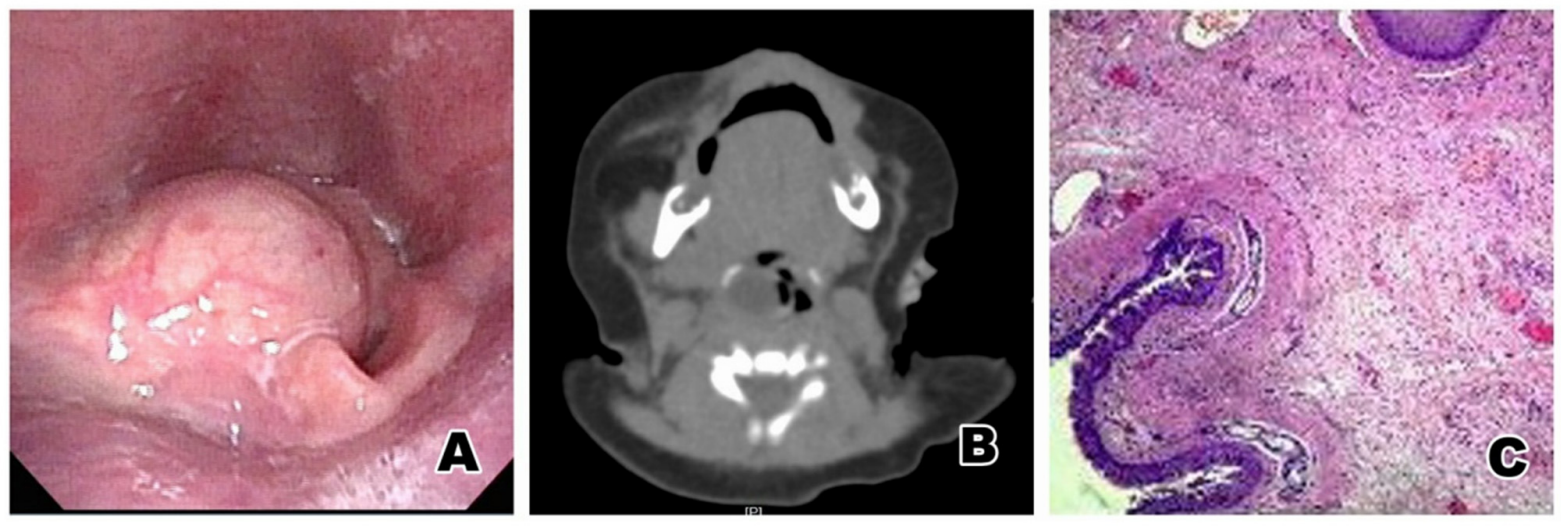
**(A)** Electronic nasopharyngoscopy before surgery: New grayish white cystic objects are found in the right epiglottis and some aryepiglottic folds, ~1.0 × 1.2 cm in size, with a smooth surface and a obscured glottis. **(B)** Pharynx CT: A low density cystic shadow can be seen on the right laryngopharynx. **(C)** Pathological results: The cyst cavity was lined with ciliated columnar epithelium, smooth muscle tissue, fibers and cartilage, with chronic inflammatory cell and lymphocyte infiltration. The pathological result was a bronchogenic cyst of the laryngopharynx.

The child was a 4-day-old boy. The child was admitted to hospital due to dyspnea and underwent mechanical ventilation for 4 days. A laryngeal mass had been found 1 day before admission, and the child had been found to have shortness of breath and dyspnea shortly after birth. The respiratory tract was cleaned, and he inhaled pure oxygen through a mask in the local neonatal department. The dyspnea did not improve, and a chest X-ray showed atelectasis on the left side. The child underwent endotracheal intubation and mechanical ventilation and was then transferred to the superior local hospital, where he underwent symptomatic treatment that included ventilator-assisted ventilation, water fasting, anti-infection, and fluid supplement. On the 3rd day, dyspnea occurred again after ventilator withdrawal, and a laryngopharyngeal mass was found during the second intubation, which is when the patient was transferred immediately to our hospital. Electronic nasopharyngoscopy showed that there were new grayish-white cystic masses in the right epiglottis and in some aryepiglottic folds. They were ~1.0 × 1.2 cm in size, their surface was smooth, and they concealed the glottis (Figure 2A). CT examination revealed a laryngopharyngeal cyst and pneumonia and showed that the upper trachea was compressed and narrowed, and the bilateral main bronchi and the bronchi in all the lobes of the right lung were narrowed (Figure 2B). Closer examination revealed no congestion in the pharynx. The palatine uvula was centered, and bilateral tonsil I° was found. After a satisfactory level of general anesthesia was achieved, it could be seen under a self-retaining laryngoscope that there were new grayish-white cystic objects in the right epiglottis and some of the aryepiglottic folds. The size was ~1.0 × 1.2 cm. The mass was punctured, and then ~2 ml of white viscous liquid was extracted. Part of the capsule wall of the pharyngeal cavity was cut off with a plasma knife, and the bleeding was stopped. The pathological diagnosis was a bronchogenic cyst (Figure 2C). The child was followed-up for 3 years after the operation, and there was no recurrence of the cyst.

### Case 3

**Figure 3:**
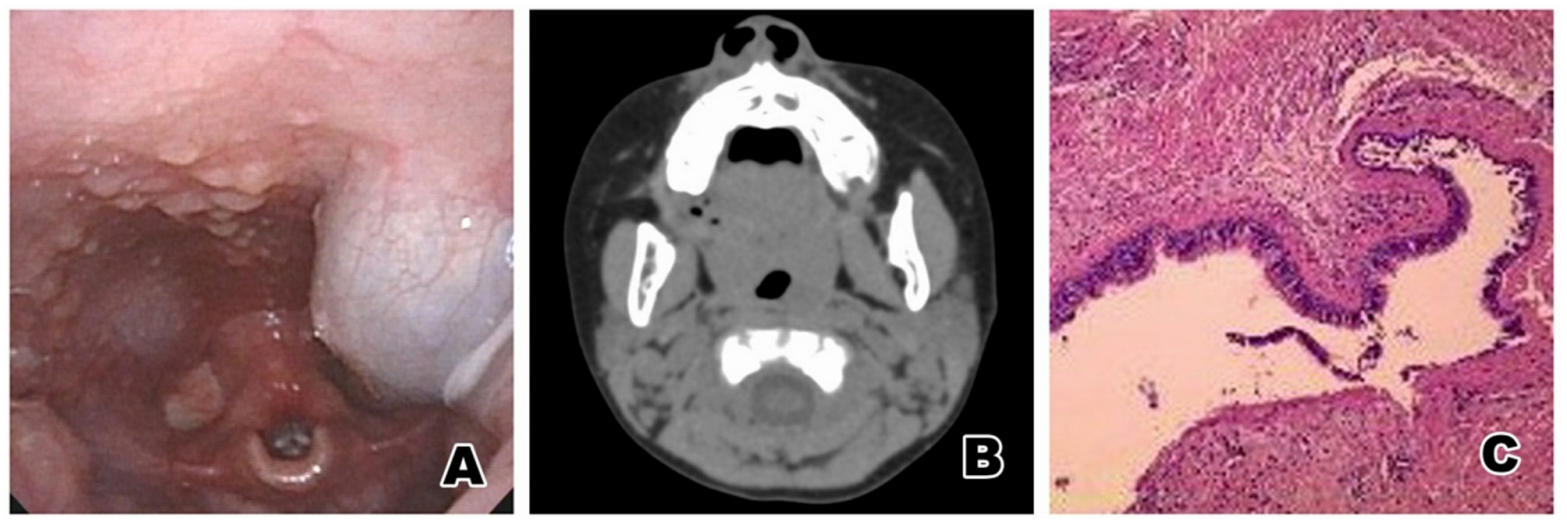
**(A)** Electronic nasopharyngoscopy before surgery: A cystic mass with a smooth surface and a size of ~1.2 × 1.3 cm can be seen in the lateral wall of the posterior pharynx of the left palatopharyngeal arch. **(B)** Pharynx CT: a left parapharyngeal quasi-round space-occupying lesion. The left piriform recess becomes shallow, a liquid low-density shadow can be seen in it, and the gas and liquid are balanced. **(C)** Pathological results: The cyst cavity was lined with squamous epithelium and pseudostratified ciliated columnar epithelium, and salivary glands, smooth muscle, cartilage tissue and lymphocyte infiltration were seen under the epithelium. The pathological result was a bronchogenic cyst of the oropharynx.

The child was a boy aged 2 years and 2 months. The child was admitted to hospital due to the discovery of a new eustachian object 2 months earlier. Close examination showed a new grayish-white object with a smooth surface, which was the size of a broad bean, located at the posterior left tonsil. Electronic nasopharyngoscopy revealed a cystic mass with a smooth surface and a size of ~1.2 × 1.3 cm in the lateral wall of the posterior pharynx of the left palatopharyngeal arch (Figure 3A). CT examination revealed a left parapharyngeal quasi-round space-occupying lesion; the left piriform recess was shallow, a liquid low-density shadow could be seen in it, with a balance of gas and liquid (Figure 3B). After a satisfactory level of general anesthesia was achieved, an opener was used, and a catheter was inserted through the double inferior meatus of the nose and through the nasopharynx. The mass was then taken out via the oral cavity, while the soft palate was pulled up to enlarge the cavity of the pharynx. Endoscopic examination showed that the base of a brown cystic mass with a smooth surface, which was ~1.2 × 1.3 cm in size, was located in the lateral wall of the posterior pharynx of the left palatopharyngeal arch. The mass was punctured, and then ~1 ml of brown viscous secretion was extracted, after which the volume of the mass had decreased significantly. Part of the capsule wall of the pharyngeal cavity was cut off with a plasma knife, and the bleeding was stopped. The pathological diagnosis was a bronchogenic cyst (Figure 3C). The child was followed up for 4 years after the operation, and no recurrence was found.

## Discussion

### Characteristics of the Disease

Bronchogenic cysts are caused by the migration and development of epithelial cells separated from the tracheobronchial tree during embryonic development. They can be classified into one of three types according to the location of the lesion: intrapulmonary, mediastinal, and ectopic. The disease can occur in all ages, and while the pulmonary type and mediastinal type are common, the ectopic type is rare. Nevertheless, in the present study, all 13 children had ectopic bronchogenic cysts, which can occur in the neck, subcutaneous space, pericardium, retroperitoneal space, intraspinal cavity, and elsewhere (2–10). Cuypers et al. reported in their study that the age of onset of bronchogenic cyst could be from 20 to 50 years (11) whereas Jiang et al.'s results suggested that there were two peaks in the age of onset: children under 6 years of age (12/16 children) and adults from 20 to 70 years of age (22/23 adults). The mediastinal type was the main type in adults, while children usually developed the disease in the neck (12). Turkyilmaz et al. reported in their study that mediastinal paraesophageal bronchogenic cysts were more common in women (65.2%), and most of them were in the middle-aged (13). Wang et al. found that intraspinal bronchogenic cysts were more common in females, and most of the patients were children and adolescents (14). The results of Lee et al.'s study showed the following: bronchogenic cysts in the neck and head were more common; most of the patients were women; the age of onset was mostly between 18 and 64 years of age; the minimum age was 3 years old; and, the disease occurred in the hyoid bone in the patient (15). In the present study, all of the patients were children, and all of them were boys. The age of onset of most of them was <6 years, the minimum age being 4 days, and only one child was over 6 years, and he was aged 6 years and 6 months. This is consistent with the results reported by Gao et al. (16), Lardinois et al. (17), and Aktogu et al. (18).

### Clinical Manifestations

A bronchogenic cyst of the pharynx is a congenital disease, of which the clinical manifestations are varied and atypical. The disease is usually asymptomatic in clinical practice, and most occurrences are found during a physical examination. The severity of the symptoms varies according to the location, size, and complications of the cyst. There are a few reports of cases of abscess, severe ecphysesis, or respiratory distress (19, 20), which are related to secondary infection, hemorrhage, rupture, and compression of the surrounding tissue. Abnormalities can be found in an imaging examination, but the diagnosis of a bronchogenic cyst depends on a pathological examination. In the present study, the cyst was found in eight children during a physical examination, and five patients visited the doctor with different degrees of upper airway obstruction. The symptoms were nasal congestion, snoring, and dyspnea. One child had dyspnea shortly after birth, and a laryngopharyngeal mass was found during a second intubation. Three children, who had progressive dyspnea and snoring, underwent electronic nasopharyngoscopy. The results revealed masses at the root of the tongue and epiglottis valley. One child, who snored at night with buccal respiration, underwent electronic nasopharyngoscopy, which revealed a cystic mass in the eustachian cushion of the nasopharynx. The severity of the symptoms, especially the obstruction of the upper airway, was related to the size and location of the mass.

### Imaging Data

Imaging examinations, such as CTs and MRIs, play an important role in the diagnosis and treatment of bronchogenic cysts. They can be used to determine the site of the disease, scope of the disease, and presence of invasion into the surrounding structures to provide a basis for the formulation of an operation plan (21–23). However, an imaging examination is not specific, and, therefore, a pathological diagnosis is still needed. Enhanced CT of a typical bronchogenic cyst can reveal their size and whether they are single-chambered, round or quasi-round, a fluid cyst, a gas cyst, a fluid gas cyst or multiple cysts, or low-density lesions with a clear boundary. The capsule wall may be thin and even, and enhancement is not often seen, or the wall can be thickened and enhanced when the cysts are infected, and cysts containing gas can be diagnosed. When the lesion is large, it pushes the surrounding tissues and organs, and the boundary with the structure of the adjacent tissue becomes fuzzy. However, some bronchogenic cysts are not connected with the trachea and bronchus, most of the fluid in the cyst is mucus, and the cyst is a gasless cyst. For example, in the present study, the cystic fluid was viscous, and, therefore, it was difficult to differentiate the disease from other diseases by utilizing preoperative imaging examination alone. MRI is superior to CT in depicting anatomical relationship and cyst definition (24). On MRI, the disease usually manifests as a round-shaped cystic mass, which is sometimes similar to soft tissue signals. Depending on the composition of the sac fluid, multiple signals could be displayed. Typically, T1WI is slightly higher than the cerebrospinal fluid signal, T2WI is equal to or higher than the cerebrospinal fluid signal, and DWI may have different performances depending on the properties of the cyst fluid. The cyst wall can be slightly strengthened, the lesion boundary clear, and the signal inside mostly uniform, but in a considerable number of cases the signal is uneven. The cause may be high protein content or bleeding in the cyst fluid.

### Pathology

Pathological examination is the gold standard for diagnosis of bronchogenic cysts, and three pathological criteria must be met: first, the cyst must have an overlying smooth muscle layer; second, it must contain epithelium derived from the foregut; and lastly, it must be attached to part of the foregut tissue. Pathological examination reveals that bronchogenic cysts occur in one or more tissues of the respiratory system, including the ciliated columnar epithelium, smooth muscle, cartilage, and fibrous connective tissue (2–4, 25, 26).

### Differential Diagnosis

Bronchogenic cysts are rare, and the misdiagnosis rate is high. The differential diagnosis subjects include intraoral thyroglossal cysts, branchial cleft cysts, dermoid cysts and epidermoid cysts, lymphangiomas, hemangiomas, teratomas, and cystic neuromas; in other words, bronchogenic cyst diagnosis involves the exclusion of many other cystic pharyngeal masses. Preoperative imaging examinations and, if necessary, enhanced CT or MRI can be used to initially determine the nature of the cyst and to understand the relationship between the cyst and the relevant surrounding tissues in order to determine the appropriate surgical method. The final diagnosis, however, requires a pathological diagnosis. (2–4, 9, 12, 13).

### Treatment

The treatment of patients with a bronchogenic cyst depends on their clinical symptoms. Unless the risk of surgery is so high that it is unacceptable, regardless of how old the patient is, the symptomatic cyst should be removed. As a result of hemorrhage, infection in the cyst or cyst enlargement compressing adjacent organs, patients with a bronchogenic cyst will have corresponding clinical symptoms, and a malignant transformation may occur. Therefore, it is suggested that the bronchogenic cyst should be resected in the early stage to relieve the clinical symptoms and prevent complications (27–29). The size and location of the lesion and its relationship with the surrounding organs will determine the specific operation procedure, but it will usually involve the complete excision of the cyst so as to avoid recurrence and other complications. However, tight adhesion to, or fusion of, the cyst and the surrounding soft tissue is a common cause of incomplete resections. Of the 13 cases in the present study, the lesion was located at the lateral pharyngeal walls in eight children, at the root of the tongue in three children, at the epiglottis and arytenoepiglottic folds in one child, and at the eustachian cushion in the final child. The cysts were closely adhered to or fused with the surrounding tissue. Therefore, the operation plan was to partially excise the capsule wall protruding into the cavity of the pharynx but to retain the deep capsule wall connected to the nasopharynx, oropharynx, and hypopharynx cavities. During the operation, the first step was to extract the fluid from the capsule to reduce the pressure, which makes it easier to explore the base. Retaining the deep capsule wall can reduce the deep trauma and bleeding caused by complete resection, and significantly reduces the operation time, thereby increasing the safety of the operation. Furthermore, a plasma knife can be used to ablate the capsule wall to prevent recurrence. After the operation, with the release of the contents of the capsule, the pressure on the surrounding tissue is relieved, and the wound margin of the capsule wall is epithelized. The residual cavity forms a shallow depression after tissue remodeling, makes the deep cavity completely open, and reduces the formation of tissue scars, and, therefore, good therapeutic effects are achieved. In the present study, all 13 children had a bronchogenic cyst in the pharynx, and all the cysts protruded into the cavity of the pharynx. They all underwent a partial excision of the mass, and the effect was satisfactory.

Our study indirectly supports the hypothesis of embryo exfoliation and translocation, in which it is proposed that in the process of embryo development, during embryo abscission and migration, the embryo is planted on the surface of the original pharynx when passing through the original pharynx cavity, forming a superficial bronchogenic cyst in the pharynx.

### Prognosis and Follow-up

The disease has a good prognosis and a low recurrence rate (30). After the operation, follow-up should be carried out, and the presence of any recurrence can be determined by physical examination, nasopharynx fibrolaryngoscopy, CT, or MRI. In summary, bronchogenic cyst is a rare cyst of the head and neck. Pharyngeal bronchogenic cysts are most common in the oropharynx. As the clinical manifestations of a bronchogenic cyst in the pharynx are varied, and the age of onset of the disease is relatively young, it merits closer attention. Imaging examination is very important, as it is helpful for cyst diagnosis and positioning. Since surgery is the most effective way to treat a bronchogenic cyst, once one is found, it should be operated on as soon as possible to avoid serious complications, such as airway obstruction caused by cyst hyperplasia. In the present study, all the children underwent partial resection of the mass, and good curative effects were achieved, which supports surgical treatment of the disease. The study also indirectly suggests that a bronchogenic cyst in the pharynx is rare, and no fistulas or sinuses were found. However, only a small number of cases were included in this study, and, therefore, the findings need to be confirmed by conducting a study on a larger number of cases in the future.

Patients with no clinical symptoms of pharyngeal bronchogenic cyst should not be treated, but patients with clinical symptoms and no obvious contraindications to surgery should be treated as soon as possible.

## Conclusion

A bronchogenic cyst is a rare cyst of the head and neck, and the most common site of the cyst is the oropharynx. The impact on airway obstruction depends on the location and size of the cyst. CT scanning is of great significance for diagnosis. Surgical treatment should be carried out as soon as possible after diagnosis, as surgery is the most effective way to treat a bronchogenic cyst, and follow-ups should be carried out regularly to prevent cyst recurrence.

## Data Availability Statement

The original contributions generated for the study are included in the article/supplementary material, further inquiries can be directed to the corresponding author/s.

## Author Contributions

YX and DS: conception and design of the research. XN and JZ: critical revision of the manuscript for intellectual content. XN: writing of the manuscript. SW: obtaining financing. LJ: statistical analysis and acquisition of data. DS: analysis and interpretation of the data. All authors contributed to the article and approved the submitted version.

## Conflict of Interest

The authors declare that the research was conducted in the absence of any commercial or financial relationships that could be construed as a potential conflict of interest.
